# Geographic variation in thermal sensitivity of early life traits in a widespread reptile

**DOI:** 10.1002/ece3.4956

**Published:** 2019-02-14

**Authors:** Brooke L. Bodensteiner, Daniel A. Warner, John B. Iverson, Carrie L. Milne‐Zelman, Timothy S. Mitchell, Jeanine M. Refsnider, Fredric J. Janzen

**Affiliations:** ^1^ Department of Ecology Evolution and Organismal Biology Iowa State University Ames Iowa; ^2^ Department of Biological Sciences Virginia Polytechnic Institute and State University Blacksburg Virginia; ^3^ Department of Biological Sciences Auburn University Auburn Alabama; ^4^ Department of Biology Earlham College Richmond Indiana; ^5^ Department of Biology Aurora University Aurora Illinois; ^6^ Department of Ecology Evolution and Behavior University of Minnesota Saint Paul Minnesota; ^7^ Department of Environmental Sciences University of Toledo Toledo Ohio

**Keywords:** *Chrysemys picta*, geographic variation, local adaptation, painted turtle

## Abstract

Taxa with large geographic distributions generally encompass diverse macroclimatic conditions, potentially requiring local adaptation and/or phenotypic plasticity to match their phenotypes to differing environments. These eco‐evolutionary processes are of particular interest in organisms with traits that are directly affected by temperature, such as embryonic development in oviparous ectotherms. Here we examine the spatial distribution of fitness‐related early life phenotypes across the range of a widespread vertebrate, the painted turtle (*Chrysemys picta*). We quantified embryonic and hatchling traits from seven locations (in Idaho, Minnesota, Oregon, Illinois, Nebraska, Kansas, and New Mexico) after incubating eggs under constant conditions across a series of environmentally relevant temperatures. Thermal reaction norms for incubation duration and hatchling mass varied among locations under this common‐garden experiment, indicating genetic differentiation or pre‐ovulatory maternal effects. However, latitude, a commonly used proxy for geographic variation, was not a strong predictor of these geographic differences. Our findings suggest that this macroclimatic proxy may be an unreliable surrogate for microclimatic conditions experienced locally in nests. Instead, complex interactions between abiotic and biotic factors likely drive among‐population phenotypic variation in this system. Understanding spatial variation in key life‐history traits provides an important perspective on adaptation to contemporary and future climatic conditions.

## 1 INTRODUCTION

Adaptation (Baumann & Conover, 2011; Kawecki & Ebert, 2004), phenotypic plasticity (Ballen, Shine, & Olsson, 2015; Charmantier et al., 2008), or some combination of the two (Crispo & Chapman, 2010; Scheiner, 2016) allow species to match phenotypes to local environments. However, species with broad geographic distributions are comprised of populations that experience different climates and thus are expected to match phenotypes to local environmental conditions across their ranges (e.g., Chevin, Lande, & Mace, 2010; Du, Warner, Langkilde, Robbins, & Shine, 2010). Assuming negligible countervailing gene flow and genetic drift, patterns of intraspecific variation among populations can shed light on microevolutionary potential in response to environmental change (Coulson et al., 2017).

Latitudinal clines are, by first impressions, evidence of local adaptation (sensu Endler, 1986). These clines are concordant among many species, leading evolutionary ecologists to devise explanations (e.g., Bergmann's Rule) for such covariances of key population traits and environmental factors (reviewed in Futuyma, 1998). Over the last century, scientists have created a series of rules to describe geographic patterns of variation in biological diversity, body shape, body size, and other such phenomena (Mayr, 1956; Rohde, 1992; Stevens, 1989). The expectation is that the basis for these rules ultimately derives from processes associated with phenotypic variability, species ranges, and biodiversity that are thermally limited. For species that are challenging to subject to particular methods of evaluating local adaptation (e.g., breeding designs in a lab setting), assessing covariances of key population traits with well‐accepted environmental proxies, such as latitude, or with actual measures of local environmental conditions, such as annual minimum temperature, is thus a crucial tool for evolutionary ecologists. Indeed, latitudinal gradients, frequently used as a proxy for temperature, have been good predictors for various phenotypic traits for many organisms across latitudinal clines (Iverson, Balgooyen, Byrd, & Lyddan, 1993; Ashton, 2002; Ashton & Feldman, 2003; Lewis, Iverson, Smith, & Retting, 2018; but, for example, see Angielczyk, Burroughs, & Feldman, 2015). For instance, in the northern hemisphere, lower latitudes are characterized by a longer growing season due to a longer duration of warmer temperatures, relative to higher latitudes (Conover & Present, 1990; Frenne et al., 2013; Worthen, 1996). Hence, latitude is frequently used as a predictor of macroclimatic conditions, and therefore as a predictor of phenotypic variation found in species with broad latitudinal ranges (Ashton, 2002; Du et al., 2010; Stinchcombe et al., 2004). Still, to what extent these macroclimatic proxies are good predictors of spatially varying phenotypic patterns within different taxa is an open question that is increasingly critical to address in light of predicted changes in climate (IPCC, 2014).

How will organisms with environmentally sensitive traits persist under rapidly changing environmental conditions? All biochemical reactions are thermally dependent, but ectotherms are well known for having an inordinate fraction of their biology linked to prevailing thermal conditions (Angilletta 2009). Many of their demographic vital rates and life‐history traits are influenced by temperature‐dependent physiological processes, for example, metabolic rates and digestive efficiency (Huey, 1982; Peterson, Gibson, & Dorcas, 1993; White, Phillips, & Seymour, 2006), behavior (Keogh & DeSerto, 1994; Mori & Burghardt, 2001; Schieffelin & de Queiroz, 1991), and development (Birchard & Deeming, 2004; Bronikowski, 2000; Gangloff, Vleck, & Bronikowski, 2015), including temperature‐dependent sex determination (TSD) in some species (Bull, 1983; Harrington, 1967; Holleley et al.., 2015; Janzen & Paukstis, 1991). In many cases, free‐living adult ectotherms alter habitat use to achieve improved thermal conditions for these traits (e.g., Sunday et al., 2014). Yet, the close connections between temperature and physiology are especially apparent during the non‐motile egg phase of embryonic development, particularly in species lacking post‐parturition parental care (Refsnider & Janzen, 2010; Shine, Elphick, & Harlow, 1997; Telemeco et al., 2016). These early life thermal vulnerabilities—and eco‐evolutionary solutions to resolve or minimize them—can perhaps be most readily revealed by examining multiple populations in taxa with broad geographic distributions (e.g., Tesche & Hodges, 2015).

**Figure 1:**
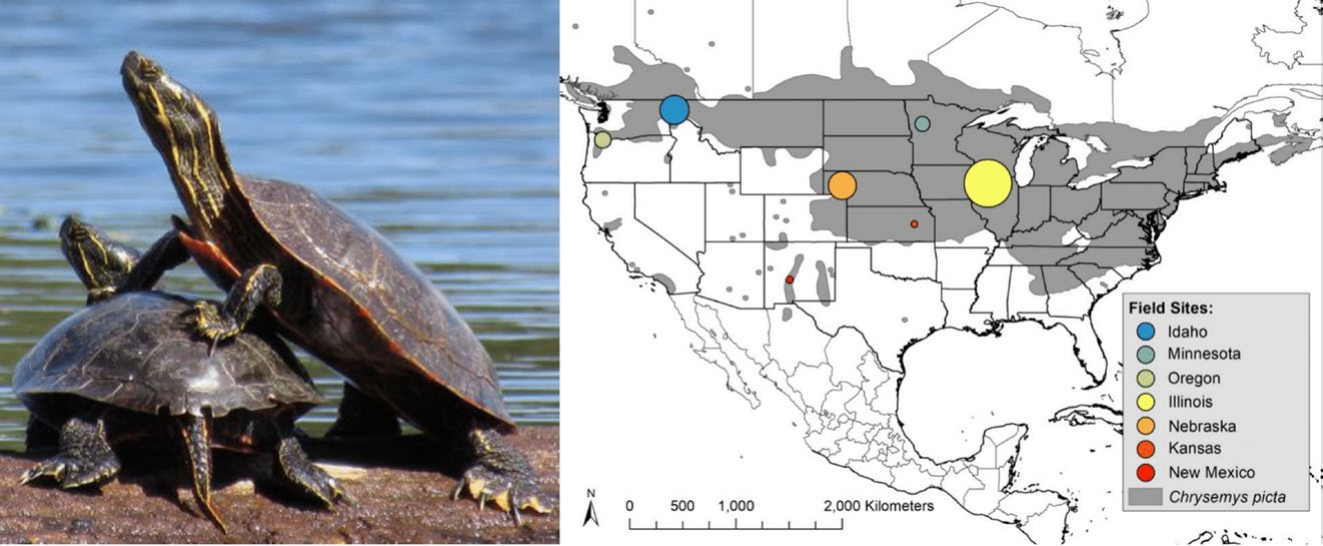
Painted turtles (*Chrysemys picta*) from the Illinois study location (left). Geographic range of painted turtles (gray). Colored circles indicate the seven study locations from which eggs were collected. The sizes of the circles are proportional to the number of offspring measured (right)

Based on its geographic, life‐history, and physiological characteristics, the painted turtle (*Chrysemys picta*; Emydidae; Figure 1) offers an excellent system for investigating fundamental questions about thermal sensitivities of early life stages in relation to macroclimatic proxies and climate change. Consequently, we compared phenotypes of developing *C. picta* from seven locations (in Idaho, Minnesota, Oregon, Illinois, Nebraska, Kansas, and New Mexico) in a common‐garden laboratory experiment. We examined how variation in key early life traits was distributed across 14 degrees of latitude and tested whether macroclimatic proxies (latitude, temperature) could predict patterns of this phenotypic variation. In particular, we focused on four embryonic traits (development rate, body size, morphological abnormalities, and hatching success) that are thermally dependent to different degrees in these turtles. Although *C. picta* has TSD (Bull, 1983), that trait was not an emphasis of this study.

**Figure 2:**
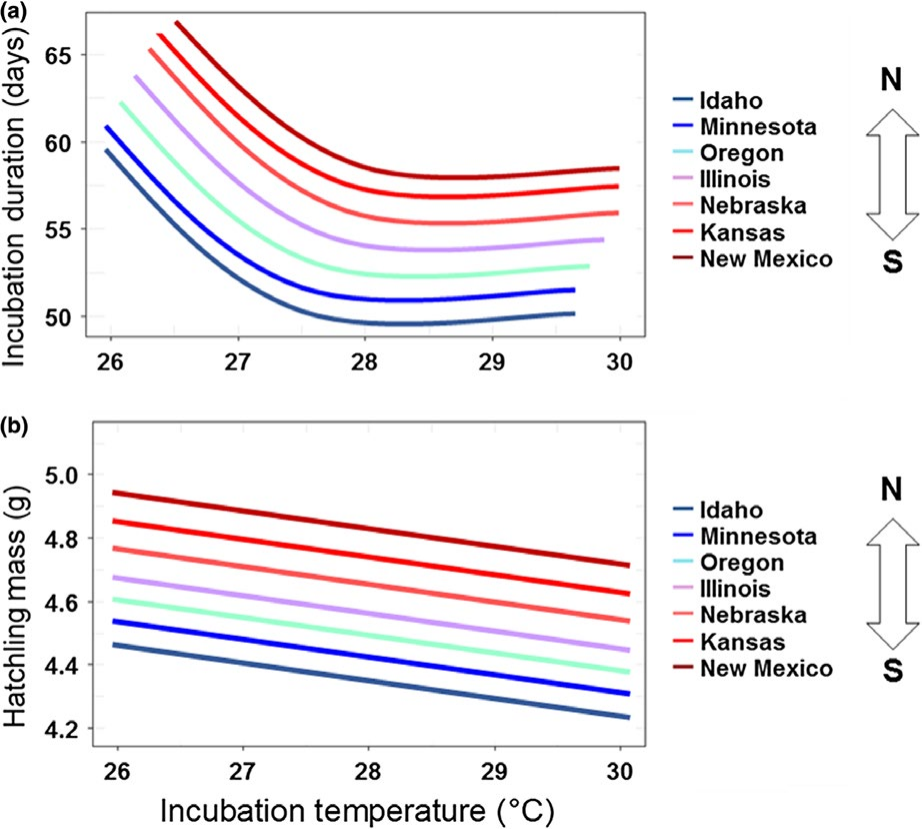
Schematic of predicted results for (a) incubation duration and (b) hatchling mass of painted turtle eggs incubated at a range of constant temperatures and sourced from seven populations spanning a 14° latitudinal gradient

Based on the literature, we had general expectations of patterns for our focal traits. We hypothesized that turtle embryos from higher‐latitude locations experience cooler nest temperatures in accord with well‐known latitudinal clines in air temperatures (www.ncdc.noaa.gov). Therefore, we predicted that eggs from northern populations would exhibit accelerated development rates, as an adaptation to potential constraints of a shorter growing season, compared to eggs from more southern locations, when incubated under constant conditions (sensu Ewert, 1985; Du et al., 2010). Emydid turtles (including *C. picta*) exhibit an intraspecific pattern following Bergmann's Rule whereby adult females at higher latitudes are larger (Ashton & Feldman, 2003; Tesche & Hodges, 2015) and produce larger clutch sizes of smaller eggs (Iverson et al., 1993). Therefore, because egg size is a major determinant of offspring size in turtles (e.g., Mitchell, Warner, & Janzen, 2013), we predicted that hatchlings from higher latitudes would have smaller body sizes than conspecifics from lower latitudes (Figure 2).

The primary period of organogenesis for *C. picta* occurs during the middle of embryonic development (Cordero & Janzen, 2014), which roughly corresponds to July in the wild, as our study populations tend to initiate and terminate the nesting season around the same time (Janzen et al., 2018; Morjan, 2003; Refsnider, Milne‐Zelman, Warner, & Janzen, 2014). Therefore, we also predicted that *C. picta* from lower latitudes (i.e., experiencing warmer temperatures) would exhibit fewer morphological abnormalities (e.g., additional scutes) and increased hatching success when incubated at higher temperatures (sensu Telemeco, Warner, Reida, & Janzen, 2013). Overall then, in northern locations we expect (a) faster development and (b) smaller hatchling size at all temperatures, and (c) more abnormalities and (d) lower hatching success at high temperatures. In sum, investigating the effects of developmental temperatures on phenotypes across multiple geographically widespread populations allows us to examine how organisms are successful across greatly diverged contemporary climates in light of a macroclimatic proxy.

## 2 METHODS

### 2.1 Study organism

The painted turtle (*Chrysemys picta*) occurs in freshwater habitats across North America (Ernst & Lovich, 2009; Starkey et al., 2003). In late spring and early summer, females emerge from the water and construct shallow, subterranean nests, typically two times each reproductive season (range 1–5), with two clutches per season considered the norm. Nests contain 1–23 eggs, with a mean of 12 eggs (Ernst & Lovich, 2009). Under field conditions, incubation typically lasts 55–85 days, with variation attributed primarily to temperature (Ernst & Lovich, 2009; Refsnider, 2016).

### 2.2 Field and lab methods

We studied distinct populations from seven geographic locations across the species range for comparison, three of these locations being the focus of long‐term research (Iverson & Smith, 1993; Janzen, 1994; Morjan, 2003; Figure 1). In late spring and early summer, we collected eggs from nests laid within 24 hr of oviposition at the field sites (9–16 clutches per location) and transported them to Iowa State University for a common‐garden incubation experiment. We weighed eggs, individually labeled them, and randomly assigned them to incubation temperature treatments from 26 to 30°C (Table 1), a range of temperatures that spans from the upper limit of viability in fine decrements to the middle of viable constant incubation temperatures (Ewert, 1979, 1985). Additionally, these temperatures fall within the range of nest temperatures documented at the Minnesota, Illinois, Nebraska, and New Mexico locations (Refsnider et al., 2014; Bodensteiner et al., unpublished). These fluctuating nest temperatures translate readily to our chosen constant incubation temperatures. For example, the typical constant temperature equivalent (essentially how physiological effects of fluctuating temperatures in turtle nests translate to those of constant temperatures; Georges, Beggs, Young, & Doody, 2005) for *C. picta* nests at the Illinois location is 28.5°C (Telemeco, Abbott, & Janzen, 2013). Thus, although those nests averaged 23.9°C with a daily range of 5°C (Refsnider et al., 2014), the physiological effects on *C. picta* embryos of that thermal regime reflect those of 28.5°C, which is in the center of our chosen series of laboratory incubation temperatures.

**Table 1:** Schematic of experimental design, with gray cells denoting successful incubation and hatching of painted turtle eggs at a given temperature and location combination

Incubation Temperatures	26°C	27°C	27.5°C	28°C	28.5°C	29°C	29.5°C	30°C
Locations								
Idaho								
Minnesota								
Oregon								
Illinois								
Nebraska								
Kansas								
New Mexico								

Note

White cells indicate temperature–location combinations that we were unable to explore.

We split eggs from a clutch across treatments to account for potential maternal effects. Incubation temperature and substrate water potential (−150 kPa) remained constant throughout development, with water added weekly to the vermiculite to maintain moist hydric conditions, which are important for proper development (Bodensteiner, Mitchell, Strickland, & Janzen, 2015; Gutzke & Packard, 1985). We incubated eggs in REVCO BOD50 environmental chambers, rotated egg boxes twice weekly to minimize impacts of within‐chamber thermal gradients, and monitored temperatures with a Thermochron iButton contained within an empty egg box in the center of each chamber (±0.2°C) (e.g., St. Juliana, 2004). Toward the end of incubation, we checked eggs daily for pipping (rupturing of the eggshell) and hatching. Once a hatchling fully emerged from its egg, we weighed it, took linear measurements (midline carapace length, carapace width, and midline plastron length), and noted any shell abnormalities, such as additional scutes (Telemeco, Warner et al., 2013). These measurements were taken for each individual. We also tallied abnormalities for advanced embryos that did not hatch.

### 2.3 Statistical methods

We conducted all statistical analyses in SAS (SAS Institute, Cary, NC) using generalized linear mixed models (MIXED procedure) to compare turtle phenotypes among locations and incubation temperatures. We ran independent models for each of the response variables: incubation duration, hatchling mass, carapace length, carapace width, and plastron length. The models contained Location (Loc) + Incubation Temperature (*T*) + Location × Incubation Temperature (Loc × *T*) + Incubation Temperature^2^ (*T* ^2^), with initial egg mass (IEM) as a covariate, and the random effect of clutch. We included the quadratic term, *T* ^2^, in our models because temperature and development are related nonlinearly (Janzen, 1993; Sokal & Rohlf, 1981). We also initially investigated sex as a potential fixed effect impacting offspring phenotypes of interest, but it was not an important factor and therefore was excluded from all analyses. Additionally, because of unbalanced groups, we used the Satterthwaite degrees of freedom correction (Littell, Stroup, Milliken, Wolfinger, & Schabenberger, 2006).

We performed statistical analyses in a two‐step process. Initially, we employed a mixed‐effect model using restricted maximum likelihood with clutch nested within Loc as a random effect to address the influence of temperature and location on the phenotypes of interest. Overall, we treated *T*, *T* ^2^, and IEM as continuous fixed effects and Loc as a categorical fixed effect. For the second step of our analytical process, we reran these models with latitude (Lat) as a continuous fixed effect in place of Loc. To further assess potential among‐location variation in thermal sensitivity, we used the model above, while restricting the data set to 27.5 and 28.5°C, temperatures at which all seven locations were represented (Table 1).

In addition to the continuous dependent variables described above, we tested for differences in hatching success and frequency of abnormalities as a function of Loc and *T*. In both cases, we used a binomial logistic regression with a logit‐link function: *Y* ~ Loc + *T* + Loc × *T* and the random effect of clutch, with the Satterthwaite degrees of freedom correction (Littell et al., 2006). Kansas was removed from the analysis of hatching success due to high egg mortality, potentially because some females were induced with oxytocin to lay these eggs before they were ready, a method not used for any other location. We used this method for turtles from Kansas, because we were unable to detect nests at this location.

## 3 RESULTS

All traits varied under common‐garden conditions in the laboratory as a function of the location of origin and the thermal incubation environment experienced. Most eggs hatched (641 out of 891; excluding Kansas, 614 out of 744), and approximately 24% of offspring exhibited scute and tail abnormalities (162 out of 665). Incubation duration declined as a function of *T* and ranged from 42 to 67 days, with a mean of 55 days. IEM positively and strongly predicted all four measures of body size (*R* ^2^ values ranging from 0.40 to 0.72). Regardless, measures of body size varied considerably. Hatchling mass ranged from 2.90 to 6.94 g, with a mean of 4.85 g. The carapace length of hatchlings ranged from 20.89 to 26.40 mm, with a mean of 25.84 mm. Plastron length and carapace width were less variable measures, ranging from 19.18 to 28.81 mm (mean 24.47 mm) and 17.10–26.00 mm (mean 23.10 mm), respectively. Consequently, these results indicate considerable scope for detecting within and among location and thermal environment sources of phenotypic variation.

### 3.1 Incubation duration

**Figure 3:**
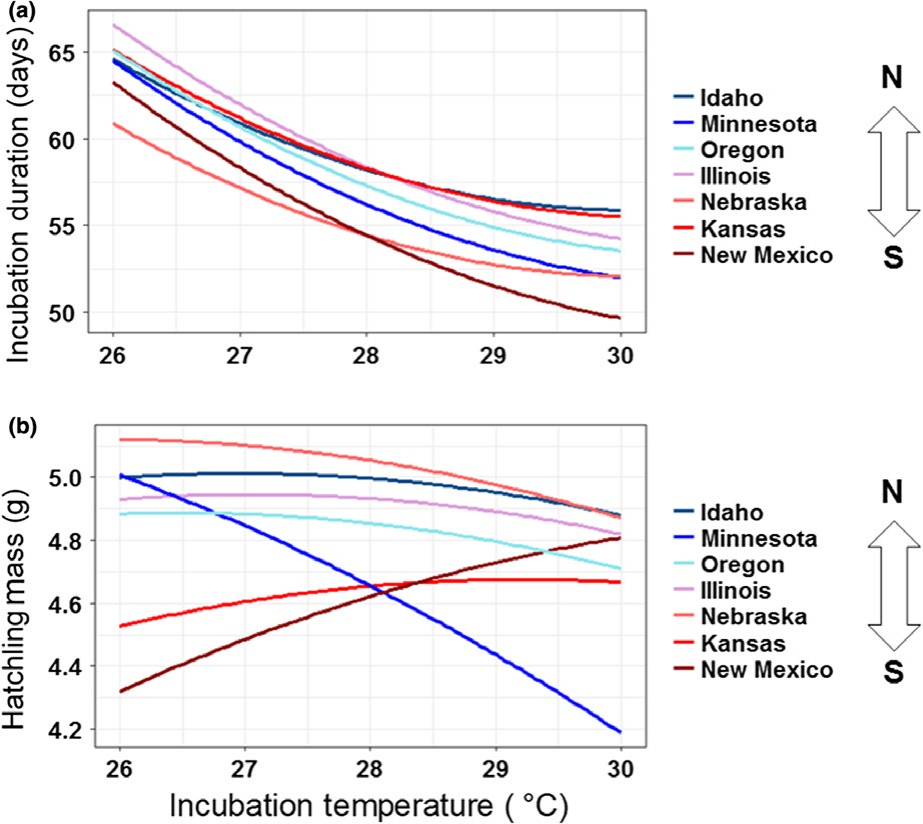
Model responses for (a) incubation duration and (b) mass of painted turtle hatchlings from a given location and incubation temperature, with initial egg mass as a covariate. Raw data not plotted to preserve clarity given the number of locations presented in each plot

Incubation duration varied among locations. As anticipated, incubation duration declined with increasing *T* and *T* ^2^; moreover, embryos from different locations responded differently to *T* (Table 2), with embryos from New Mexico and Nebraska exhibiting especially fast developmental rates at any given *T* (Figure 3). Lat was also a statistically significant predictor when substituted into the model for Loc (Table 2). Embryos from higher Lat generally took longer to develop at any given *T*.

**Table 2:** Two‐step process for mixed models evaluating incubation duration (days), hatchling mass (grams), and linear measurements (millimeters) of hatchling painted turtles (see text for details)

	Location descriptor	Temperature	Temperature^2^	Temp * Location	Initial Egg Mass
Step 1, Location as category					
Incubation duration	*F*_6,601_ = 11.99 ***p* < 0.0001**	*F*_1,609_ = 117.39 ***p* < 0.0001**	*F*_1,609_ = 97.80 ***p* < 0.0001**	*F*_6,574_ = 11.28 ***p* < 0.0001**	*F*_1,639_ = 3.21 *p* = 0.0736
Hatchling mass	*F*_6,608_ = 5.11 ***p* < 0.0001**	*F*_1,630_ = 2.97 *p* = 0.0853	*F*_1,630_ = 3.19 *p* = 0.0744	*F*_6,587_ = 5.16 ***p* < 0.0001**	*F*_1,634_ = 907.76 ***p* < 0.0001**
Carapace length	*F*_6,604_ = 0.49 *p* = 0.8150	*F*_1,650_ = 6.04 ***p* = 0.0142**	*F*_1,650_ = 6.22 ***p* = 0.0129**	*F*_6,594_ = 0.54 *p* = 0.7773	*F*_1,479_ = 183.53 ***p* < 0.0001**
Plastron length	*F*_6,598_ = 1.35 *p* = 0.2343	*F*_1,646_ = 4.91 ***p* = 0.0270**	*F*_1,646_ = 5.09 ***p* = 0.0244**	*F*_6,584_ = 1.24 *p* = 0.2845	*F*_1,544_ = 267.69 ***p* < 0.0001**
Carapace width	*F*_6,600_ = 1.59 *p* = 0.1472	*F*_1,648_ = 1.85 *p* = 0.1748	*F*_1,648_ = 2.00 *p* = 0.1577	*F*_6,591_ = 1.54 *p* = 0.1622	*F*_1,431_ = 82.63 ***p* < 0.0001**
Step 2, Location as latitude					
Incubation duration	*F*_1,611_ = 12.14 ***p* = 0.0005**	*F*_1,613_ = 116.56 ***p* < 0.0001**	*F*_1,614_ = 89.27 ***p* < 0.0001**	*F*_1,581_ = 14.41 ***p* = 0.0002**	*F*_1,604_ = 7.83 ***p* = 0.0053**
Hatchling mass	*F*_1,618_ = 5.77 ***p* = 0.0166**	*F*_1,642_ = 3.38 *p* = 0.0664	*F*_1,643_ = 2.02 *p* = 0.1562	*F*_1,598_ = 5.33 ***p* = 0.0214**	*F*_1,530_ = 993.89 ***p* < 0.0001**
Carapace length	*F*_1,616_ = 0.00 *p* = 0.9849	*F*_1,659_ = 5.39 ***p* = 0.0205**	*F*_1,659_ = 6.21 ***p* = 0.0129**	*F*_1,609_ = 0.00 *p* = 0.9680	*F*_1,304_ = 226.47 ***p* < 0.0001**
Plastron length	*F*_1,611_ = 0.36 *p* = 0.5479	*F*_1,658_ = 4.83 ***p* = 0.0284**	*F*_1,658_ = 6.61 ***p* = 0.0104**	*F*_1,600_ = 0.48 *p* = 0.4879	*F*_1,353_ = 310.83 ***p* < 0.0001**
Carapace width	*F*_1,617_ = 2.61 *p* = 0.1070	*F*_1,656_ = 1.19 *p* = 0.2767	*F*_1,655_ = 2.82 *p* = 0.0937	*F*_1,611_ = 2.76 *p* = 0.0971	*F*_1,272_ = 100.06 ***p* < 0.0001**

Note

*p*‐values <0.05 are presented in bold font.

### 3.2 Hatchling body size

**Figure 4:**
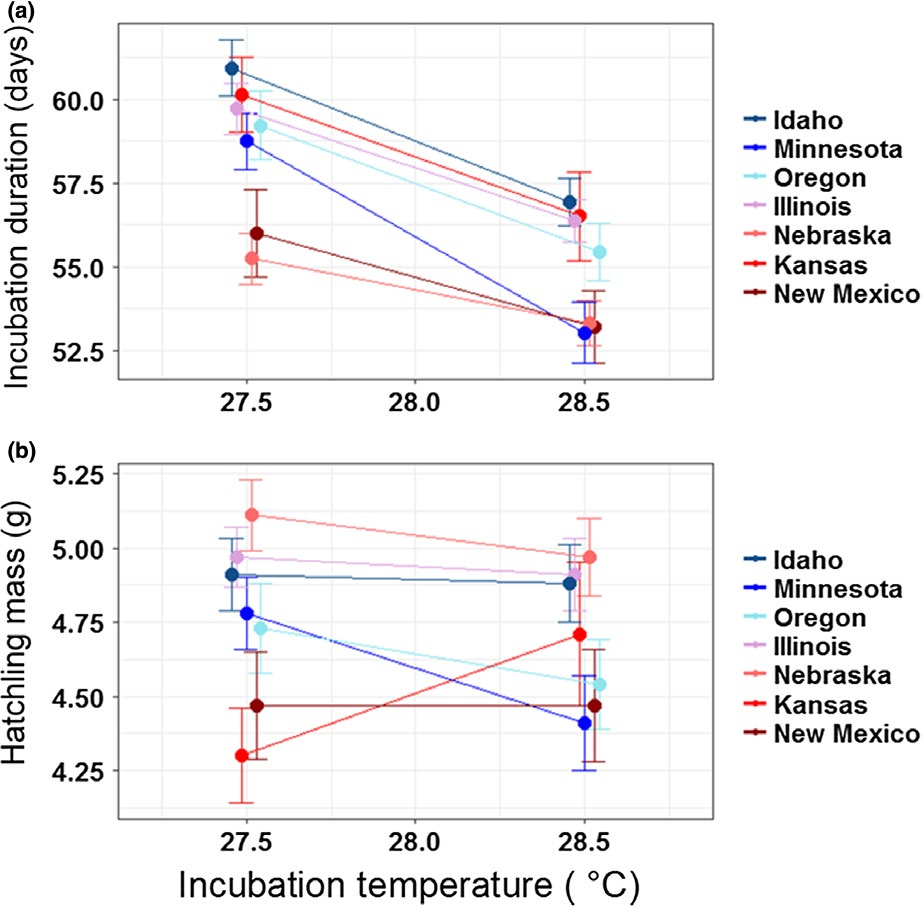
Phenotypic responses, (a) incubation duration and (b) mass, of painted turtle offspring from eggs incubated at constant 27.5 and 28.5°C. Values shown are LSM ± *SE*

Initial egg mass was the primary driver of size at hatching, positively predicting much of the variation in all four measures of body size (see above *R* ^2^ values). As for incubation duration, we detected differences in IEM‐adjusted body size among Loc (Figure 3), although this geographic variation only yielded statistically significant results for hatchling mass (Table 2). Of particular note, relative hatchling mass from Minnesota declined substantially with increasing *T* in contrast to the other locations (Figure 3). Carapace length, carapace width, and plastron length did not differ substantively with respect to Loc as a function of *T* despite evident differences (Supporting Information Figure S1 in Appendix S1). Lat was not a good predictor of IEM‐adjusted body size (Table 2), with one exception: Lat was more positively related to hatchling mass at lower *T* than at higher *T* (Figure 3).To identify whether phenotypic differences among locations were best explained by intrinsic (e.g., genetic) factors or phenotypic plasticity, we tested for differences among locations within two *T* scenarios (common‐garden conditions at 27.5 and 28.5°C where all seven locations were represented). We detected differences among all seven locations within each incubation regime (Figure 4; Supporting Information Figure S2 in Appendix S1). Notably, compared to those from other locations, embryos from Nebraska developed particularly fast and were heavier at hatching after accounting for IEM, whereas offspring from Minnesota were relatively long and wide on average (Supporting Information Figure S2 in Appendix S1).

### 3.3 Abnormalities and hatching success

For hatchling abnormalities and hatching success, we detected no effect of Loc, *T*, or Loc × *T* (all *p* > 0.30; Supporting Information Table S1 and Figure S3 in Appendix S1:). Even though Loc was not statistically significant, Oregon offspring had a substantially reduced probability of having an abnormality, whereas Illinois offspring were over 10 times more likely to have an abnormality than in Oregon (Supporting Information Figure S3 in Appendix S1).

## 4 DISCUSSION

Widespread species experience divergent macroenvironmental conditions across their ranges, and populations frequently exhibit phenotypic responses to match those environmental differences locally via some combination of adaptation and phenotypic plasticity (Ballen et al., 2015; Baumann & Conover, 2011; Charmantier et al., 2008; Kawecki & Ebert, 2004; Scheiner, 2016). Elucidating the responses of geographically widespread organisms with temperature‐sensitive traits to current thermal conditions therefore could provide insights into potential microevolutionary responses to environmental change. We found substantial variation in thermal reaction norms among our widely distributed painted turtle populations, but this variation could not be attributed entirely to latitude. In other words, this common macroclimatic proxy was ineffective in explaining observed among‐population phenotypic variation. Even so, we detected evidence under common‐garden conditions suggesting that heritable factors and/or pre‐ovulatory maternal effects contributed to location‐specific phenotypes of neonatal turtles, most notably incubation duration and hatchling mass.

Latitude, serving as a proxy for environmental conditions (especially temperature), has been used to successfully explain phenotypic variation among populations of species with broad geographic ranges (see references cited earlier). We obtained a dissimilar outcome for the traits we studied in painted turtles. We did not find support for the prediction that embryos from higher‐latitude locations would develop faster than embryos from more southern locations under common‐garden incubation conditions. We detected differences among our locations—and latitude did predict some patterns in this study—but no clear clinal pattern emerged. This unexpected result contrasts with prior work in some other reptiles. For example, the incubation periods for fence lizards (*Sceloporus undulatus*) (Du et al., 2010) and common snapping turtles (*Chelydra serpentina*) (Ewert, 1985) vary inversely with latitude, being shorter for embryos from cooler localities. Notably, however, fence lizards must grow quickly and compete for mates at early ages, similar to other short‐lived organisms, and snapping turtles emerge from nests within days of completing incubation; by comparison, painted turtle hatchlings overwinter in nests for many months before emerging, with multiple years before maturity (e.g., Spencer & Janzen, 2014). These divergent life histories could explain why we did not detect accelerated embryonic development in painted turtles from higher latitudes. Although incubation duration in painted turtles might not appear to be under strong selection, when compared to other reptile species that emerge from the nest shortly after hatching, it could be important because hatchling position within the nest might influence the ability to survive subzero winter temperatures (Colbert, Spencer, & Janzen, 2010). Even so, if painted turtles used more yolk to develop quickly (and weigh less at hatching) and remain in the nest during the warmer temperatures of late summer and fall, this could be problematic for survival during the overwintering and dispersal life stages when resources are limited until they reach the water. Such hatchlings would presumably catabolize more residual yolk and body stores due to increased metabolic expenditure at higher temperatures (Huey, 1982; Muir, Dishong, Lee, & Costanzo, 2013; Willette, Tucker, & Janzen, 2005). With ongoing climatic warming, especially during winter at northern latitudes (Liu, Curry, Dai, & Horton, 2007), current developmental life‐history strategies could become detrimental, disproportionately impacting those populations.

According to Bergmann's Rule, as environmental temperatures decrease (i.e., at higher latitudes or elevations), the animal body size increases (Mayr, 1956). This maxim was originally described for endotherms such as mammals and birds, which often follow the pattern. More recently, it has been applied to some ectothermic reptiles (Ashton, 2002; Ashton & Feldman, 2003; Ashton, Tracy, & Queiroz, 2000; Lewis et al., 2018). For example, consistent with Bergmann's Rule, mean asymptotic body size of adult turtles is larger at higher latitudes (Ashton & Feldman, 2003) and these larger body sizes are associated with variation in life‐history traits (e.g., increased time to sexual maturity and larger clutch sizes; Iverson et al., 1993). We predicted that hatchlings would follow the inverse to Bergmann's Rule because painted turtle eggs, which largely determine offspring size (see Results), are smaller at higher latitudes (Iverson et al., 1993). Our hypothesis illustrates the complex nature of selective forces differentially acting on various life stages (Sherratt, Vidal‐García, Anstis, & Keogh, 2017). In this study, the inverse Bergmann's Rule for hatchling body size was not supported. We detected no clinal variation in our measures of offspring body size, besides the effect of latitude and the interaction between incubation temperature and latitude on hatchling mass that seems to be driven by lower incubation temperatures (Figure 3; Supporting Information Figure S1 in Appendix S1). Thus, among‐location variation in microenvironmental conditions might underpin this observation rather than a macroclimatic proxy (Cooley, Floyd, Dolinger, & Tucker, 2003; Tesche & Hodges, 2015).

We predicted that eggs from the more southern populations that experience the warmest air temperatures during key stages of development would be more resilient to increased incubation temperatures, having fewer morphological abnormalities and increased hatching success relative to presumably cooler northern populations. Extreme incubation temperatures can increase probability of morphological abnormalities (Telemeco, Warner et al., 2013), which may be negatively correlated with fitness. In long‐term study populations, morphological abnormalities are present in much higher levels in young than in adults, suggesting that selection eliminates many offspring with abnormalities before they reach adulthood (Arnold & Peterson, 2002; Telemeco, Warner et al., 2013). Also, incubation temperature and hatching success are often inversely correlated, such that eggs incubated at the highest temperature are less likely to hatch than those at lower temperatures (Van Damme, Bauwens, Braña, & Verheyen, 1992; Gutzke & Packard, 1985; Packard, Packard, & Birchard, 1989). However, we found no statistically significant effect of location, incubation temperature, or their interaction on the presence of abnormalities or on hatching success. These results are somewhat surprising, especially given the notably elevated percentage of abnormal hatchlings from Illinois (Supporting Information Figure S3 in Appendix S1; Telemeco, Warner et al., 2013). In contrast to those in our other study locations, painted turtles in the Mississippi River might be exposed to higher levels of abnormality‐inducing contaminants such as PCBs (Adams, Baker, & Kjellerup, 2016).

Latitudinal gradients, driven by concordant thermal changes, underpin among‐population variation in a number of traits in a diversity of organisms. Hence, the overall absence of clinal variation in our study was unexpected. We examined substantial numbers of turtles originating from seven locations across a considerable geographic range at multiple incubation temperatures, so our inability to detect much clinal variation may not derive from insufficient statistical power (but see Gienger, Dochtermann, & Tracy, 2018). Alternative explanations should be considered. First, latitude may be an inappropriate macroclimatic proxy (Hawkins & Felizola Diniz‐Filho, 2004). For example, longitude might better predict temperature‐related offspring traits in painted turtles from our study locations, as it correlates with percent annual sunshine (e.g., Ewert, Etchberger, & Nelson, 2004). Second, we focused on thermal incubation environments toward the upper end of viable conditions (Table 1). Incubating eggs at lower temperatures (i.e., 21–25°C), which are also regularly encountered in natural nests (e.g., Mitchell, Maciel, & Janzen, 2015), instead might have elicited phenotypic patterns consistent with variation in our macroclimatic proxy and allowed a more thorough investigation of the thermal reaction norm for these phenotypic traits. Thermal incubation environments being at relatively high constant temperatures might present novel environments for development, which could elicit unexpected patterns of plasticity and genetic variation (Rose 1985). Even so, at least with respect to our Illinois location, the laboratory constant‐temperature regime nicely encompassed constant‐temperature equivalents derived from nests (Telemeco, Abbott et al., 2013). Third, we characterized thermal “performance” curves of traits for our study locations using constant temperatures that, even if standard and fairly comprehensive, conceivably might not adequately reflect phenotypic responses to the fluctuating environmental conditions usually experienced in nature (Ketola & Kristensen, 2017). For example, variance in temperatures during development impacts reptilian phenotypes (Bowden, Carter, & Paitz, 2014; Mullins & Janzen, 2006; Shine et al., 1997; Webb, Brown, & Shine, 2001), such as incubation duration under laboratory and field conditions (Ashmore & Janzen, 2003; Shine & Harlow, 1996). As the variance in temperature increases, so does the likelihood that some portion of the daily temperature dips below a lower threshold for embryonic development or exceeds a critical thermal maximum and therefore could affect rates of development and resulting phenotypic variation differentially among populations (Andrews, Mathies, & Warner, 2000; Georges et al., 2005; Telemeco, Abbott et al., 2013). In sum, in seeking to understand among‐population patterns of phenotypic variation, we should focus more attention on evaluating responses to thermal conditions experienced in nests rather than relying on macroclimatic proxies and on experiments employing relatively simplistic environmental conditions (sensu Bowden et al., 2014).

Under constant incubation at 27.5 and 28.5°C, thermal reaction norms for all traits varied among our widely distributed study locations of painted turtles. However, this phenotypic variation was not explained by our macroclimatic proxy, unlike in many systems where latitudinally linked climatic conditions accord with patterns of certain traits observed in nature (Bradshaw & Holzapfel, 2008; Coyne & Beecham, 1987; David & Bocquet, 1975). Instead, environmental aspects that better reflect microclimates experienced in nests may drive variation in phenotypic reaction norms among locations in our system, including precipitation/hydric conditions (Bodensteiner et al., 2015), nest‐shade cover (Janzen, 1994), and/or regional weather patterns (e.g., thermal variation; Vasseur et al., 2014).

Regardless of the source(s), we detected evidence consistent with genetic differentiation or preovulatory maternal effects for nearly all traits measured under common‐garden conditions. Phenotypic differences among locations often persisted even when embryos were reared under identical thermal and hydric conditions. Moreover, locations did not all respond the same way to incubation temperature (Figure 3), consistent with possible genotype by environment interactions underpinning certain phenotypes. But do these outcomes necessarily imply adaptation, especially in the absence of a predictive macroclimatic proxy? Although unlikely (e.g., see Starkey et al., 2003), reduced gene flow and genetic drift could potentially drive these population‐level phenotype patterns. Regardless, a tension between the two primary explanations for among‐population phenotypic variation is evident for other traits in turtles and suggests their coexistence and even synergy. For example, Ewert, Lang, and Nelson (2005), and Schroeder, Metzger, Miller, and Rhen (2016) provide evidence of genetically based local adaptation for thermal sensitivity of temperature‐dependent sex determination (TSD) among conspecific turtle populations arrayed from north to south in the United States, whereas Refsnider and Janzen (2012) instead detected considerable phenotypic plasticity in a common‐garden field experiment for TSD‐related nesting behavior among widely dispersed turtle populations.

One explanation for the modest geographic patterns of phenotypic differentiation found here is that nest‐site choice could buffer ambient climatic conditions via selection of specific microhabitats within each location (Bernardo, 1996; Refsnider & Janzen, 2010). This behavior substantially affects offspring phenotypes and survival in a variety of taxa (Brown & Shine, 2004; Ewert et al., 2005; Refsnider, 2016; Resetarits, 1996), especially in those that lack parental care, where developing embryos are exposed to the abiotic and biotic conditions of the environment in which they are oviposited (Mitchell et al., 2015, 2013). Nest‐site choice in painted turtles is heritable under certain environmental conditions, permitting adaptive microevolution of this trait (McGaugh, Schwanz, Bowden, Gonzalez, & Janzen, 2009). However, its potential to maximize individual fitness might derive more from plasticity in this trait (Kamel & Mrosovsky, 2006; Refsnider & Janzen, 2012, 2016). That is, plasticity in nest‐site choice could be mitigating macroclimatic differences among locations and therefore could explain why our macroclimatic proxy, generally, was a poor predictor of among‐location thermal reaction norms for phenotypic variation in painted turtle offspring.

Our study populations have had thousands of years to respond locally to differing climates post‐Pleistocene glaciation (Starkey et al., 2003). A major concern is that given the rate at which the world is changing, many long‐lived organisms like turtles may not be able to keep pace with that change (Refsnider & Janzen, 2016; Visser, 2008). This issue is especially pertinent for species with key embryonic traits intrinsically tied to temperature, as embryos cannot compensate for unfavorable circumstances. Indeed, it is imperative to understand how species accommodate different environmental conditions, whether via “direct” adaptation or through phenotypic plasticity, to gauge their vulnerability. In this experiment, we substituted space for time to gain insights into this matter. That is, at the macroclimatic level, we sought to clarify how populations in warmer locations might differ from those in cooler locations. In the absence of detecting strong evidence of phenotypic matching to macroclimatic conditions, we therefore are turning our attention to measuring in situ characteristics (e.g., nest temperatures) across space and time for these painted turtle populations. A major challenge to ecologists in this rapidly changing world is elucidating the complex interactions between abiotic and biotic factors that shape among‐population phenotypic variation.

## CONFLICT OF INTEREST

None declared.

## AUTHOR CONTRIBUTIONS

DAW and FJJ conceived and planned experiments. All authors contributed to data collection. BLB analyzed data, and BLB and FJJ wrote the manuscript. DAW, JBI, TSM JMR, and CLMZ contributed to the final version of the manuscript.

## Data Availability

Data available from the Dryad Digital Repository: https://doi.org/10.5061/dryad.8qq1607.
