# Supplementary material for: Geographic variation in thermal sensitivity of early life traits in a widespread reptile
Source: Ecol Evol. 2019 Feb 14;9(5):2791–802. doi: 10.1002/ece3.4956 (PMC6405489; doi:10.1002/ece3.4956)
Supplement: Supplementary file 1 [file ECE3-9-2791-s001.pdf]

Supplementary Appendix 1:

Table S1: Effects of location and incubation temperature on the probability of a painted turtle embryo surviving to hatch, excluding Kansas and the probability of a painted turtle hatchling exhibiting morphological abnormalities, all locations included.

	Probability of Hatching Success	Probability of Abnormalities
Temperature	$F_{1,626}$=0.11, p=0.7432	$F_{1,651}$=1.34, p=0.2476
Location	$F_{5,626}$=0.56, p=0.7275	$F_{6,651}$=0.78, p=0.5868
Temperature x Location	$F_{5,626}$=0.63, p=0.6752	$F_{6,651}$=0.78, p=0.5286

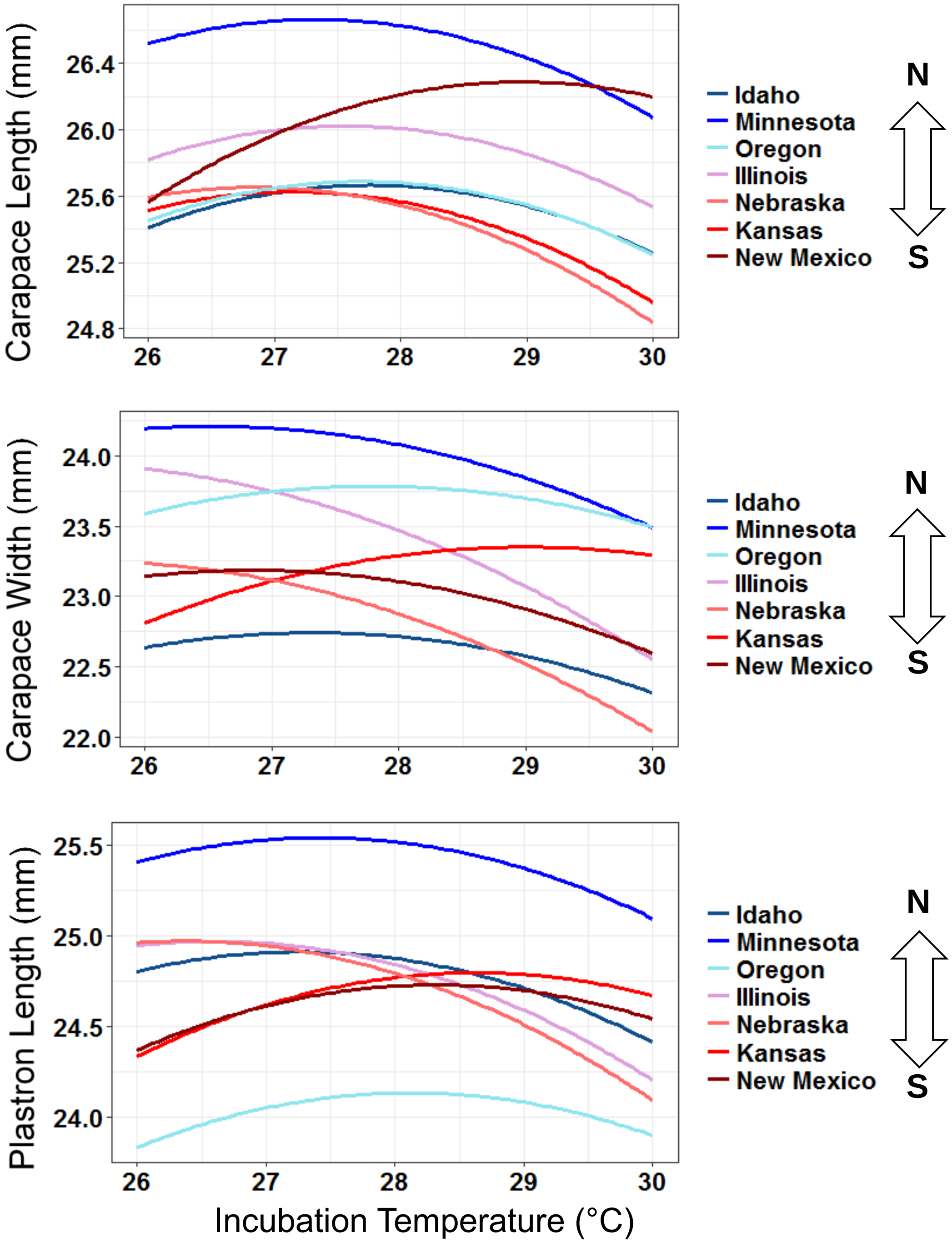

Figure S1: Model responses for carapace length of painted turtle hatchlings from a given location and incubation temperature, with initial egg mass as a covariate. Raw data not plotted to preserve clarity given the number of locations presented in each plot.

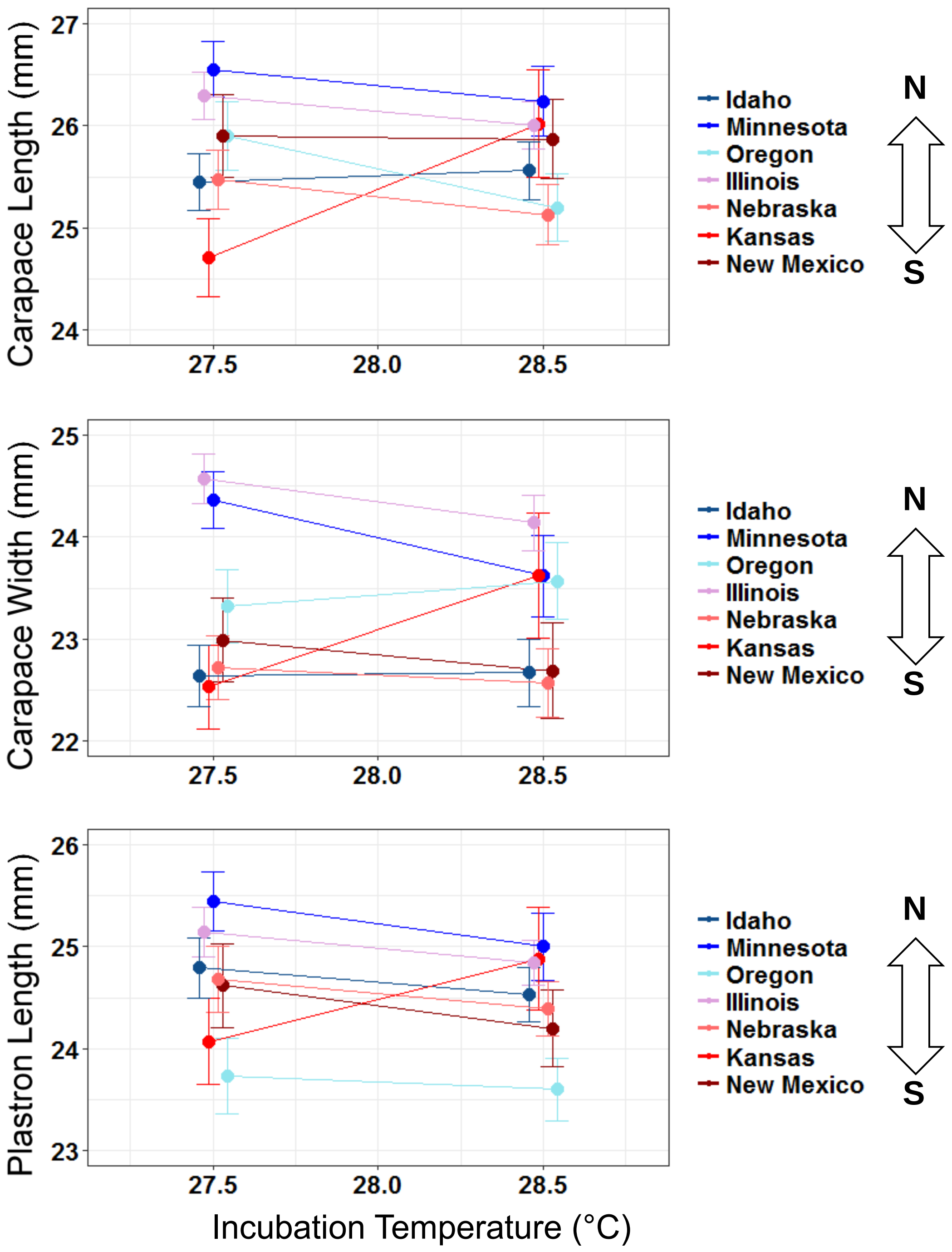

Figure S2: Phenotypic responses, carapace length, carapace width, and plastron length, of painted turtle offspring from eggs incubated at constant temperatures of 27.5 and 28.5°C. Values shown are LSM±SE.

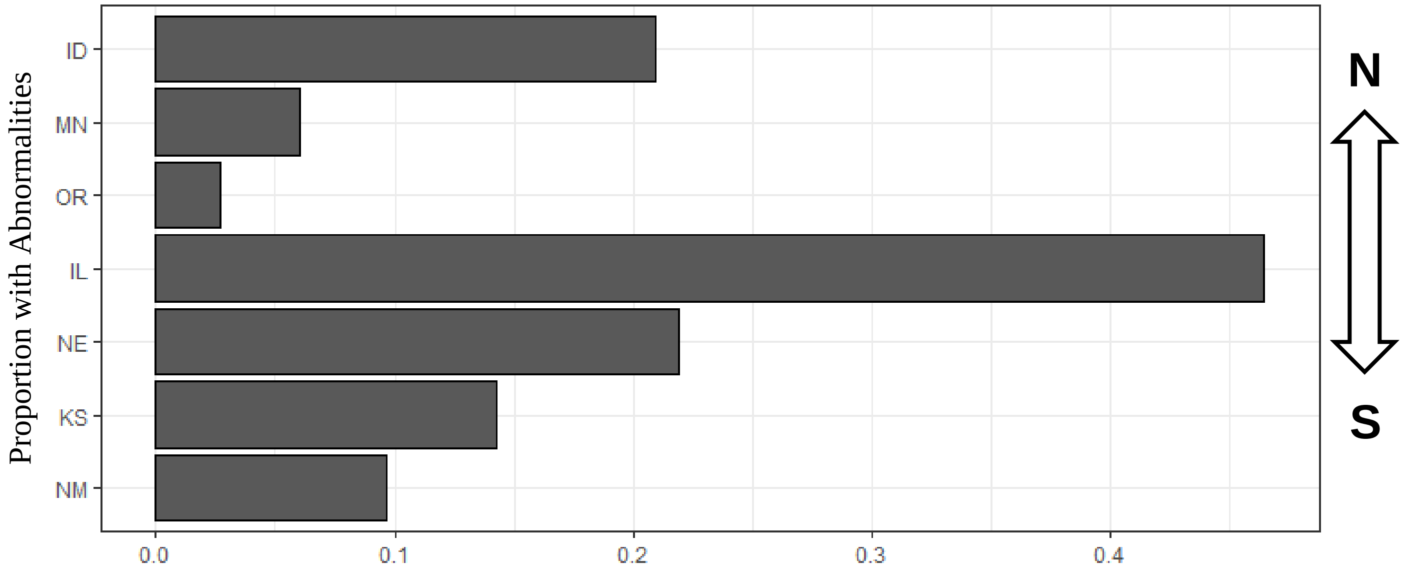

Figure S3: Proportion of painted turtle hatchlings with morphological abnormalities across locations and incubation temperatures. We detected no effect of location, temperature, or their interaction (all $P > 0.30$).

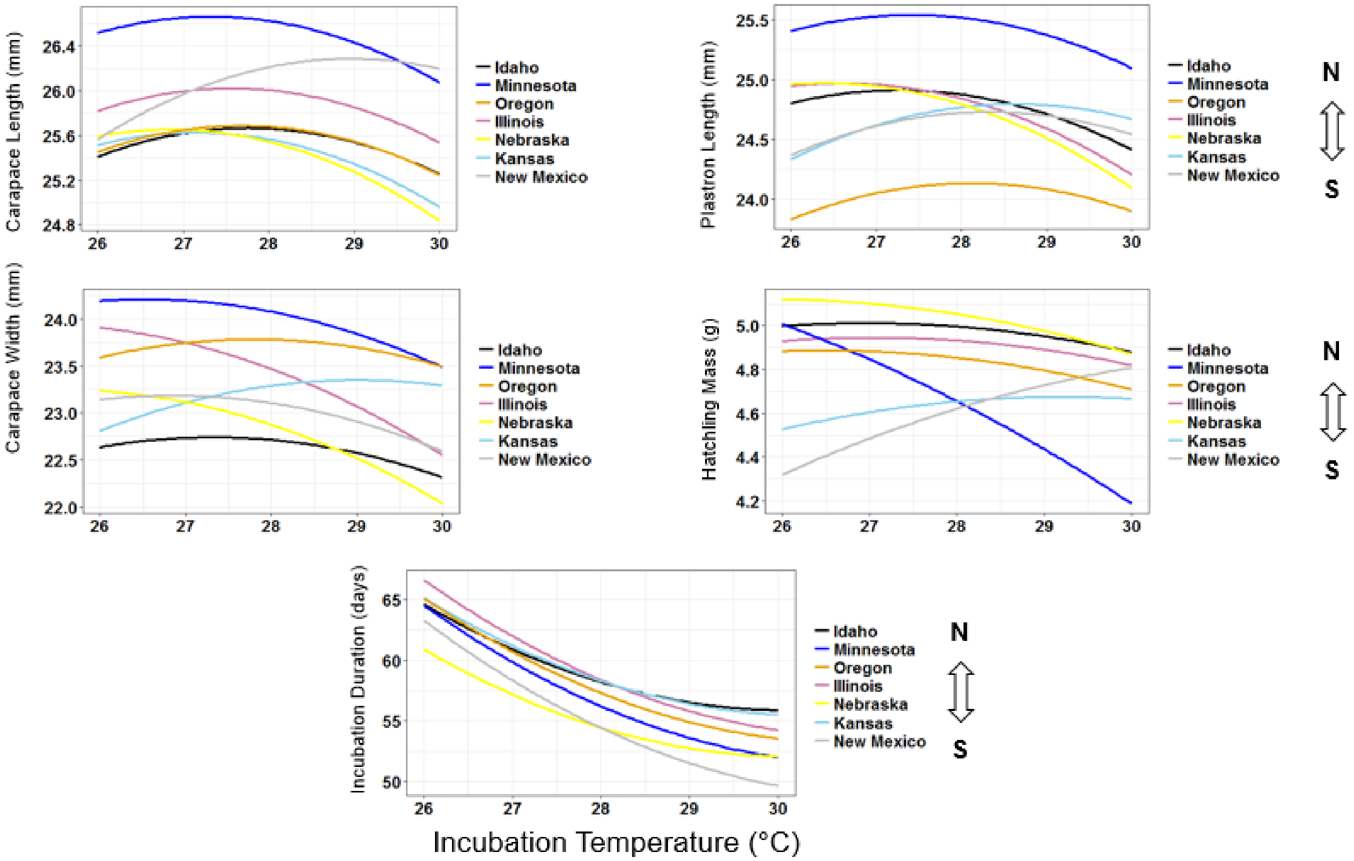

Figure S4: Model responses, using a **colorblind friendly palette**, for body size measurements and incubation duration of painted turtle hatchlings from a given location and incubation temperature, with initial egg mass as a covariate.

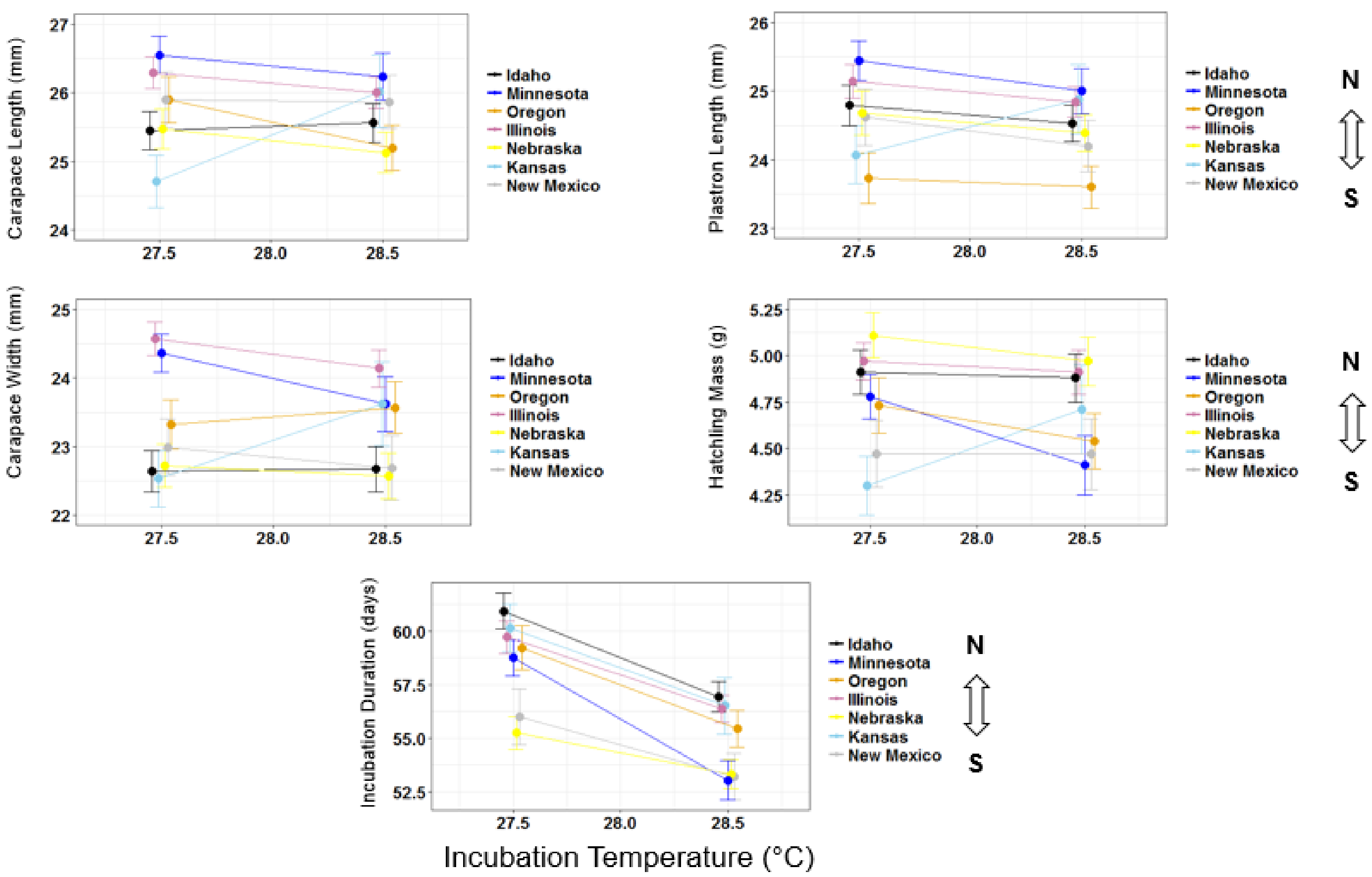

Figure S5: Phenotypic responses of painted turtle offspring from eggs incubated at constant temperatures of 27.5 and 28.5°C. Values shown are LSM±SE using a **colorblind friendly palette**.
